# An interdisciplinary team successfully treated a newborn with severe skin necrosis caused by infusion extravasation: A case report

**DOI:** 10.1097/MD.0000000000045850

**Published:** 2025-11-14

**Authors:** Danfeng Li, Mingli Jiang, Hongyan Wu, Caixiao Shi, Wenqing Kang, Yingyuan Wang, Miaomiao Shi, Guilin Fan, Yanyan Sun

**Affiliations:** a Neonatal Intensive Care Unit, Children’s Hospital Affiliated to Zhengzhou University, Zhengzhou City, Henan Province, China; b Children’s Hospital Affiliated to Zhengzhou University, Zhengzhou, Henan Province, China; c Nursing Department of Children’s Hospital Affiliated to Zhengzhou University, Zhengzhou, Henan Province, China.

**Keywords:** case report, interdisciplinary teams, NICU, skin necrosis, total parenteral nutrition

## Abstract

**Rationale:**

Critically ill neonates in the neonatal intensive care unit are at high risk of severe tissue damage from drug or total parenteral nutrition (TPN) extravasation due to their unique physiology, and traditional single-discipline management is often insufficient. This report presents a severe TPN extravasation case that was successfully treated through interdisciplinary collaboration.

**Patient concerns:**

A small-for-gestational-age infant was admitted to an external department for intractable vomiting and received TPN infusion. On the fourth day of treatment, severe fluid extravasation occurred at the puncture site on the left ankle, presenting with swelling, skin whitening, and evident tenderness. The skin broke down 6 hours later, forming a significant ulcer.

**Diagnoses:**

The injury at the left ankle puncture site was diagnosed as a severe TPN extravasation, resulting in a grade IV skin injury with a 2.5 cm × 1.6 cm ulceration reaching the subcutaneous tissue.

**Interventions:**

After transfer from an external hospital where initial saline irrigation proved ineffective, our hospital initiated a multidisciplinary team (including plastic surgeons, orthopedic surgeons, wound/ostomy nurses, and neonatal staff). The team performed 6 staged debridements to remove necrotic tissue. Wound care involved sequential application of alginate, foam, hydrophilic fiber silver-containing, and hydrocolloid dressings, supplemented with recombinant human epidermal growth factor. Multimodal analgesia included lidocaine gel, fentanyl infusion, non-nutritive sucking, and oral glucose water.

**Outcomes:**

After 27 days of treatment, the skin wounds dried and healed completely without exudation, and the patient was discharged. A follow-up 7 days after discharge confirmed complete skin healing without scarring and no limitations on physical activity.

**Lessons:**

For small-for-gestational-age infants receiving hypertonic/irritant medications like TPN, central venous access should be prioritized. It is crucial to formulate specific emergency protocols for neonatal TPN extravasation and incorporate them into nurse training. For cases of severe necrosis, interdisciplinary consultation is essential for optimal outcomes, with particular attention paid to comprehensive pain management and patient comfort.

## 1. Introduction

**Figure 1.**
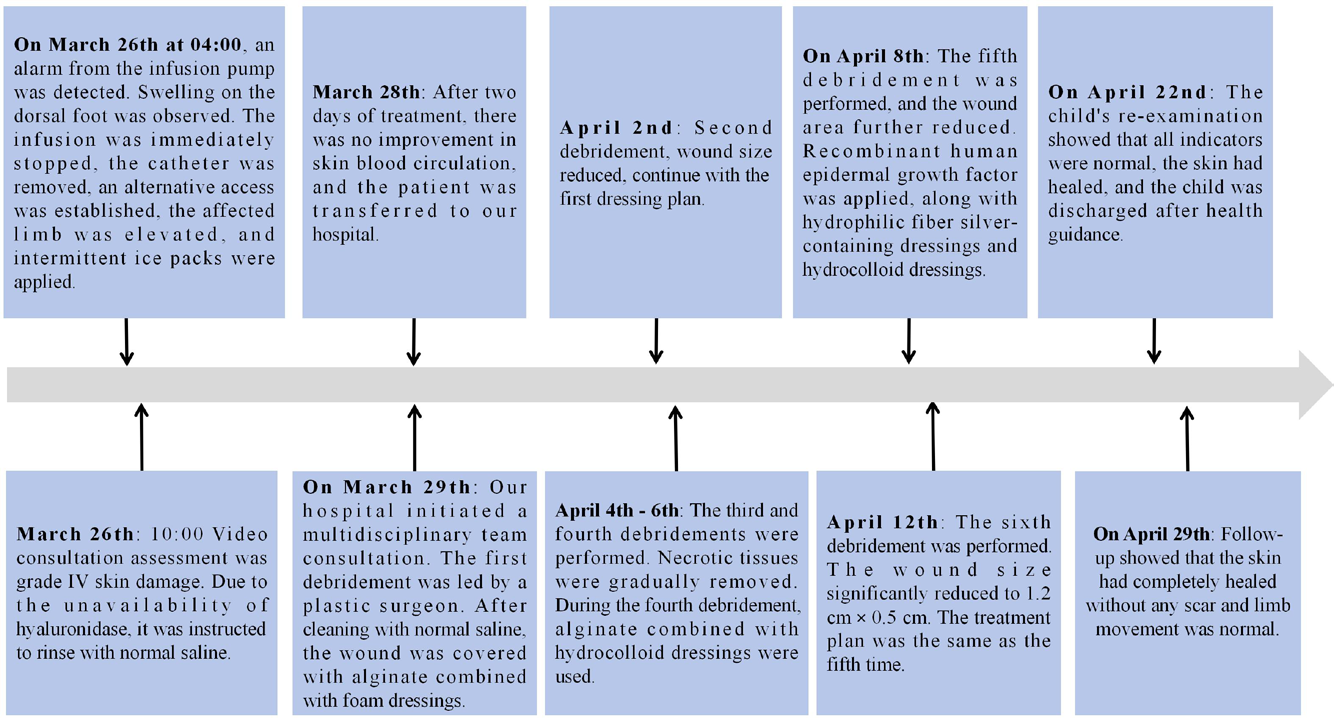
The dynamic progress trajectory of skin extravasation injury care is presented in the form of a timeline: After the child’s skin extravasation and 27 days of hospitalization, 6 sequential debridement and repair treatments were completed through the collaboration of an interdisciplinary team, and the skin was eventually healed.

Studies have shown that the proportion of critically ill newborns born in recent years is significantly greater than that in the past.^[1]^ Neonates suffering from gastrointestinal, digestive, and absorptive dysfunction often require indwelling intravenous catheters to complete drug infusion and nutritional support after admission to the neonatal intensive care unit (NICU).^[2]^ Clinical access is primarily established through the peripheral vein and central vein, among which a peripheral venous catheter is often the first choice for early diagnosis and treatment in the NICU due to its advantages of convenient operation, minimal invasion, and safety. However, newborns have special anatomical structures and physiological functions, characterized by thin subcutaneous fat, slender lumina, and thin, fragile walls, unable to verbally express pain. When peripheral veins are used for infusion, irritating liquids or drugs can easily penetrate the perivascular tissue space, thereby increasing the risk of drug extravasation.^[3–5]^ Irritant solutions include but are not limited to some antibiotics (amphotericin B, vancomycin, etc), calcium gluconate, potassium chloride, vasoactive drugs, and total parenteral nutrition (TPN). Many studies have shown that long-term infusion of hypertonic TPN is a high-risk factor for neonatal skin extravasation, especially when a central venous catheter is not used.^[6,7]^ In a study involving 1409 neonates, 2.4% of individuals suffered from severe skin extravasation, and TPN was the main type of fluid administered to these patients.^[8]^ A case report from South Korea revealed that an infant developed extravasation of TPN solution after infusion, which caused severe skin necrosis and osteofascial compartment syndrome; the patient finally underwent fasciotomy combined with skin grafting.^[9]^ This case report presents a neonate with severe skin necrosis caused by TPN extravasation. After a multidisciplinary medical team implemented a multimodal analgesic regimen and carried out standardized comprehensive treatment (wound debridement, application of alginate dressing, foam dressing, hydrophilic fiber silver-containing dressing, and hydrocolloid dressing, combined with recombinant human epidermal growth factor application). After 27 days of sequential repair, the full thickness skin of the necrotic area was finally repaired. There was no scar tissue and the limb movement was unimpaired (Fig. 1).

## 2. Timeline

The dynamic progression trajectory of skin extravasation injury care is shown in Figure 1.

### 2.1. Information on the patient

The mother of the newborn had a history of 3 unexplained abortions and delivered a healthy full-term boy by cesarean section in 2020. This was her fifth pregnancy. Except for mild anemia in the mother, the other indicators were normal. At 38^+5^ weeks of gestation, the baseline fetal heart rate was 80 to 90 beats/min, and an emergency cesarean section was performed. The birth weight of the newborn was 2.52 kg (young for gestational age), the Apgar score was 9 at 1 minute (minus points: mild cyanosis of the skin), and the score was 10 at 5 minutes. The newborn was admitted to the NICU because of neonatal vomiting due to unknown causes. Chest x-ray, routine blood examination, temporary fasting, and TPN treatment were prescribed. In the early morning of the fourth day of treatment, the nurse identified an alarm of the injection pump (pump speed of 10.5 mL/h, osmotic pressure of 860 mOsm/L) during the inspection and then found a swelling at the puncture site of the left ankle of the child. The skin at the puncture site was white, and the child complained of pain (imaging data were not retained). Under the guidance of the nurse group leader, the infusion and urethral catheter were immediately stopped, and a new venous pathway was established. Simultaneously, the limb and discontinuous ice compress processing were raised. After the above treatment was administered, the skin swelling of the ankle was not significantly relieved, and the skin was damaged 6 hours later. The nursing management team of the department contacted the intravenous therapy specialist nurses of our hospital for a video consultation. The pharmacy of the hospital where the patient was treated at that time did not have hyaluronidase available in stock. We recommended administering normal saline irrigation therapy. According to the venous extravasation injury grading scale proposed by the Infusion Nursing Society and Thigpen, the skin injury of the child was considered to be grade IV.^[10,11]^ The above information was obtained from the medical records of the transferred hospitals and the caring nurses.

### 2.2. Key information

On admission, the skin blood supply of the patient did not improve 2 days after treatment for extravasation, and, combined with the diagnosis of vomiting and skin necrosis, the patient was admitted to our hospital. Physical examination revealed that the left ankle skin was swollen, and an oval area of soft tissue necrosis on the skin surface, approximately 2.5 cm × 1.6 cm in size, was observed, extending to the depth of the subcutaneous tissue. The wound was dark red, with a large amount of exudate. Laboratory tests revealed that inflammatory marker levels were normal (C-reactive protein < 0.5 mg/L and procalcitonin within normal limits), and blood culture, liver function, kidney function, and electrolyte levels were all within the normal range. The platelet count showed significant fluctuations (581 × 10^9^/L to 723 × 10^9^/L in the early stage), considered to be related to stress from tissue damage. The persistently low red blood cell count and hemoglobin level (hemoglobin 78–88 g/L) aligned with the diagnosis of neonatal anemia. The patient was treated with intravenous infusion of ceftazidime for anti-infection, oral iron supplementation for anemia. Given the absence of thrombotic manifestations (e.g., extremity cyanosis or abnormal blood flow) despite elevated platelet levels, the anticoagulation regimen remained unchanged, with increased monitoring of peripheral circulation implemented.

## 3. Clinical data

**Figure 2.**
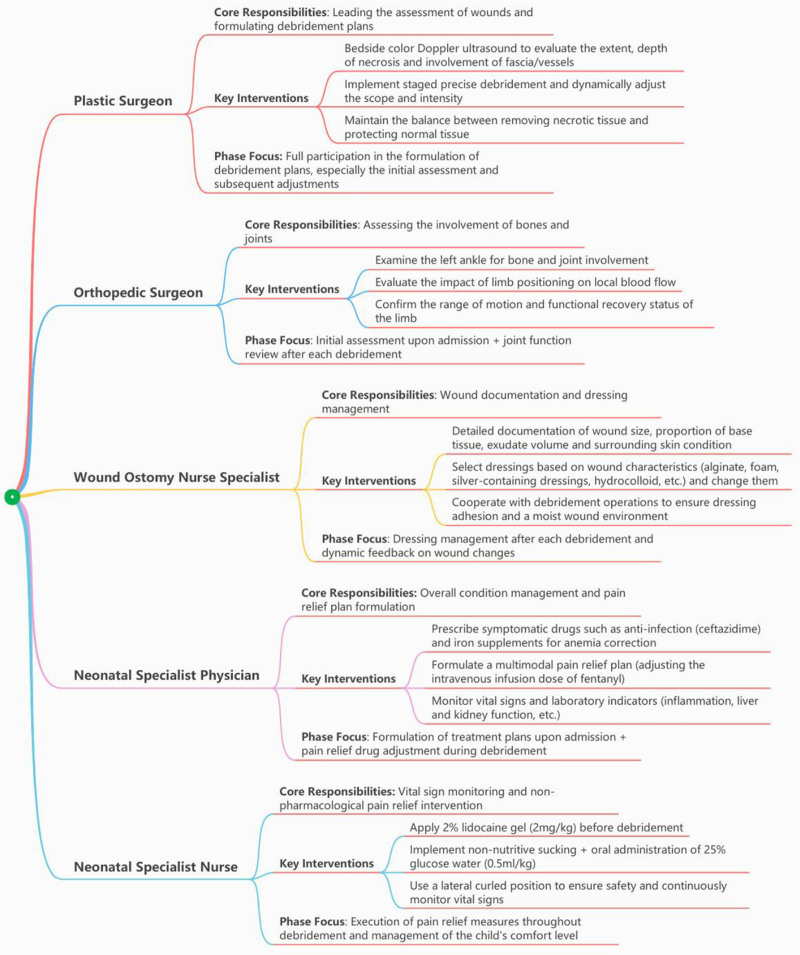
Responsibilities of the interdisciplinary team members.

After the infant patient was admitted to the neonatal intensive care unit of our hospital, he was placed on a GE Healthcare Giraffe Warmer infant radiant warmer table, wrapped in a swaddle, and the left lower limb was elevated (with the sensor of the radiant warmer table exposed). An interdisciplinary wound management team, including plastic surgeons, orthopedic surgeons, wound stoma specialist nurses, and neonatal specialist medical staff, was established. The team immediately started multidisciplinary consultation (Fig. 2). An orthopedic doctor’s ability to assess a patient at the bedside is key to determining whether subcutaneous tissue necrosis ranges, deepens, or involves the deep fascia/blood vessels, and to formulate a debridement scheme in stages. The dynamic adjustment scope of debridement and strength plays a key role in debridement operations, simultaneously ensuring the accurate removal of necrotic tissue and protecting normal tissues. An orthopedic doctor was responsible for evaluating the left leg’s bone joints involved, including whether exosmosis affected the left ankle bone or joint, and assessing whether the body’s mobility was influenced by the local blood supply. The wound stoma specialist nurses were responsible for keeping detailed records of the wound status after each debridement (including the extent of the wound, the proportion of basal tissue, the amount of exudation, and the surrounding skin condition) and for the selection and replacement of the corresponding dressing plan based on the characteristics of the wound. Neonatal specialist staff were responsible for monitoring overall vital signs, combined with the characteristics of neonatal pain and the establishment of individualized analgesic solutions: debridement by neonatal specialist nurses 15 minutes before 2% lidocaine gel (dose of 2 mg/kg) was applied to the wound,^[12]^ 5 minutes before the physician adjusted intravenous fentanyl to 1 μg/(kg h).^[13]^ Moreover, neonatologists implemented a nondrug analgesic intervention by combining non-nutritive sucking with the administration of a 25% glucose solution (0.5 mL/kg) orally, and non-nutritive sucking continued until the end of the debridement. Given that the debridement procedure may elicit a cough reflex in pediatric patients due to pain stimulation, the lateral recumbent position with flexion of the lower limbs was implemented during oral glucose administration to minimize the risk of aspiration.^[14]^

**Figure 3.**
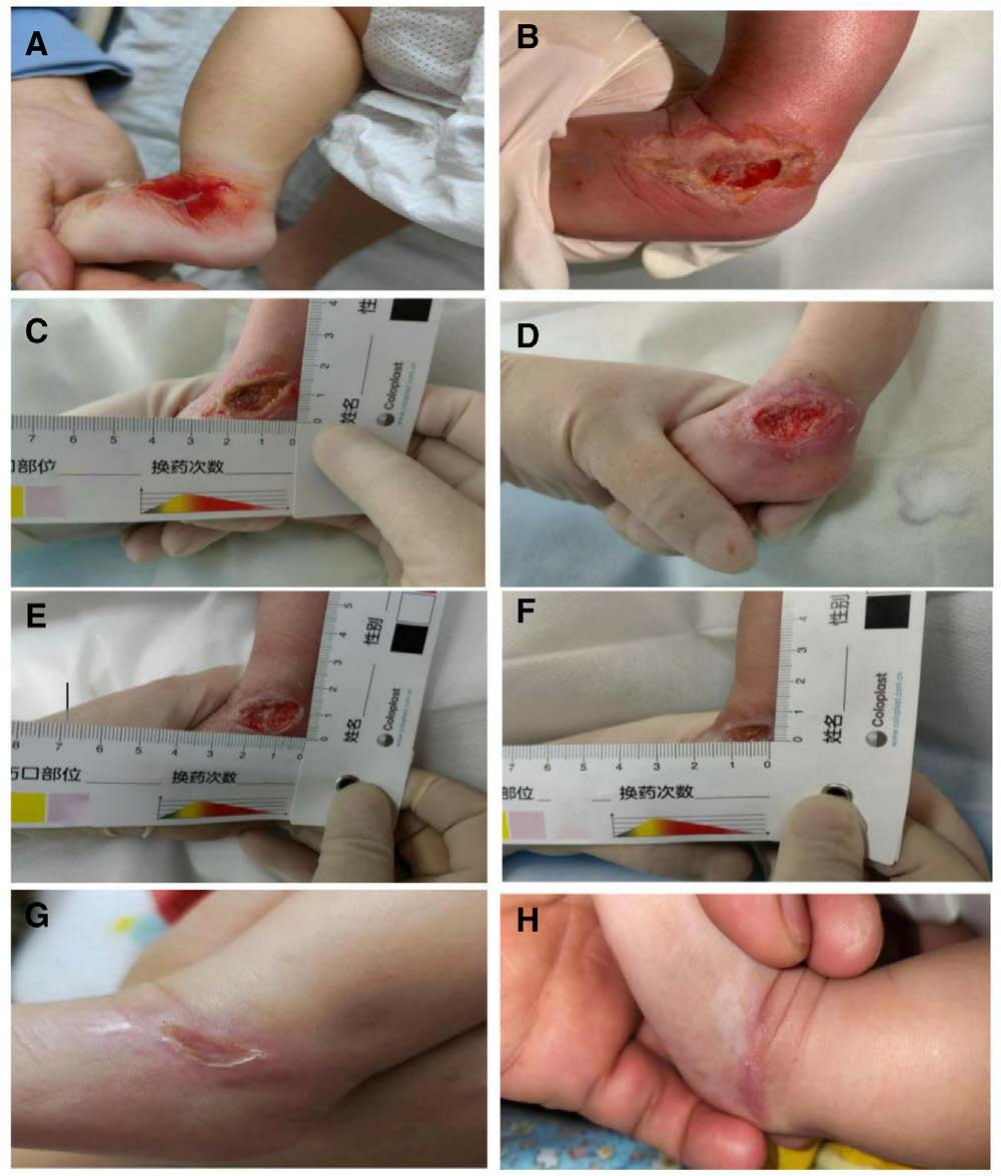
(A) A 3 cm × 2 cm edematous area is visible on the dorsal side of the left foot, with a central skin ulceration area. The ulcer surface is dark red and congested, covered with a small amount of exudate. (B) The ulcer surface measures 2.5 cm × 1.6 cm, reaching the subcutaneous tissue layer in depth. The ulcer surface is dark red, with a large amount of exudate before debridement. (C) (after the first debridement): The ulcer surface measures 2.5 cm × 1.5 cm, with the same depth as before. 10% of the ulcer surface is yellow necrotic tissue, and 90% is red granulation tissue, with a large amount of exudate. (D) (after the second debridement): The ulcer surface has shrunk to 2.3 cm × 1.5 cm, still at the subcutaneous tissue layer. 5% of the ulcer surface is yellow necrotic tissue, and 95% is red granulation tissue, with moderate exudate and maceration around the skin. (E) (after the third/fourth debridement): The ulcer surface measures 2.2 cm × 1.3 cm, with the same depth. 95% of the ulcer surface is red granulation tissue and 5% is yellow necrotic tissue, with a small amount of exudate and continuous maceration around the skin. (F) (after the sixth debridement): The ulcer surface measures 1.2 cm × 0.5 cm, with 98% covered by red granulation tissue and 2% residual yellow tissue. There is a small amount of exudate and the skin texture around has returned to normal. (G) The ulcer surface is dry and scabbed, without exudate, entering the healing stage. (H) The skin has healed without scar formation, and there is no hindrance to limb movement.

After the first mechanical debridement, the wound size was 2.5 cm × 1.5 cm, which was the depth of the subcutaneous tissue layers. After assessment, the wounds contained 10% yellow necrotic tissue, 90% red granulation tissue, a large amount of drainage, and a dip in the surrounding skin (Fig. 3C). The treatment method was as follows: after the wound was cleaned with normal saline, it was covered with an alginate dressing and a foam dressing. After the second mechanical debridement, the wound narrowed to 2.3 cm × 1.5 cm, and the depth was still within the subcutaneous tissue layer. Necrotic tissue accounted for 5% of the slope of the drainage quantity of the medium (Fig. 3D), and the debridement dressing scheme was extended for the first time. After the third mechanical debridement, the wound size did not change significantly, but the necrotic tissue was completely removed, and the amount of exudate decreased. The treatment plan of the first debridement was still being used. After the fourth mechanical debridement, the wound size decreased to 2.2 cm × 1.3 cm, the depth still reached the subcutaneous tissue layer, the proportion of necrotic tissue was higher than that before 2 days, and the amount of exudate decreased (Fig. 3E). For this treatment, normal saline was first used to clean the wound, and then an alginate dressing combined with a hydrocolloid dressing was applied to cover the wound. After the fifth mechanical debridement, the wound was further reduced to 1.8 cm × 1.0 cm. The depth was still within the subcutaneous tissue layer, but it was shallower than before, and the proportion of necrotic tissue remained at 5%. The treatment measures were as follows: after the wound was cleaned with normal saline, epidermal growth factor was applied, and then a hydrophilic silver-containing dressing combined with a hydrocolloid dressing was used to cover the wound. After the sixth mechanical debridement, the wound size was reduced to 1.2 cm × 0.5 cm, with a small amount of necrotic tissue remaining. The color and texture of the surrounding skin were normal, and the wound boundary was clear (Fig. 3F). No peculiar smell was found in the wound throughout the debridement cycle.

The neonatal pain, agitation and sedation scale (N-PASS) is suitable for assessing acute and persistent neonatal pain and postoperative and mechanical ventilation-related pain.^[15]^ The N-PASS was recommended by the American Academy of Pediatrics and the Neonatologists Branch of the Chinese Medical Doctor Association^[16,17]^; it can comprehensively evaluate neonatal pain in 6 dimensions. The N-pass is practical and feasible for application in clinical practice. In this study, the N-PASS score was independently completed by neonatal specialist nurses who had received standardized training on the Chinese version of the N-PASS scale. The total score of the N-PASS remained at 2 from the first to the fourth debridement, primarily due to the scores of the facial expression and limb movement dimensions, each of which was 1 point. During the fifth and sixth debridement, the total score decreased to 1, which manifested as a slightly frowning facial expression. Throughout the debridement process, the child’s vital signs remained stable, and no adverse reactions to the pain were observed.

### 3.1. Follow-up results

After 27 days of extravasation injury, the skin wound on the left ankle had dried without exudation and healed well (Fig. 3G). Before discharge, the doctor in charge instructed that compound polymyxin ointment, combined with recombinant human epidermal growth factor, should be applied 3 times a day. Health education was also provided to the child’s family members at discharge to inform them about the pharmacological characteristics of the drug and the specific method of use. After 7 days of outpatient follow-up, the skin texture of the child returned to normal, the thickness of the subcutaneous tissue evaluated by ultrasound was not different from that of the contralateral side, and the active range of motion of the limb was similar to that found in children of the same age (Fig. 3H).

## 4. Discussion

The extraction of intravenous fluids refers to the clinical complication of unplanned infiltration of corrosive fluids into extravascular tissues. The incidence of venous extravasation is high. Without timely intervention, it can lead to progressive damage from local inflammation to tissue necrosis.^[18]^ A prospective study involving 452 peripheral intravenous catheters in 152 infants in the Department of Neonatology of a hospital in Turkey reported that the Incidence of severe skin extravasation was 11.7%.^[19]^ Multivariate analysis confirmed that the risk of severe skin extravasation was significantly related to the child (body weight, infection, and disease), puncture catheter (puncture site, puncture time, and retention time), hyperosmotic drug (including but not limited to calcium, potassium, TPN, etc), and excessive infusion volume per unit time.^[5,20]^ TPN is a high-risk drug for extravasation injury in children.^[21]^ In our study, the patient required TPN infusion due to disease-related factors, which is consistent with the conclusion that hypertonic drugs significantly increase the risk of extravasation in previous studies. The high osmolality of TPN, combined with small-for-gestational-age and relatively little subcutaneous fat, further aggravates the risk of extravasation injury. Therefore, individualized protection strategies should be developed for children at high risk in clinical practice, such as prioritizing central venous access to reduce the stimulation of hypertonic fluid on peripheral blood vessels, strictly controlling the infusion speed and concentration of TPN solution, and strengthening the dynamic monitoring and evaluation of the infusion site to detect and address the risk of extravasation in time.

In the clinical management of fluid extravasation, patients with grades I and II disease are typically treated with conservative observation, whereas patients with grades III and IV disease require active intervention. Specific treatment options include the use of normal saline solution for wound irrigation and the application of hyaluronidase to promote drug diffusion. For patients complicated with infection or tissue necrosis, surgical treatment,^[22]^ such as abscess cavity drainage and necrotic tissue debridement, should be performed. Several studies have shown that the intervention program of hyaluronidase sealing combined with normal saline irrigation has significant clinical efficacy.^[21,23]^ Hyaluronidase can specifically degrade hyaluronic acid in the extracellular matrix, significantly improving the penetration efficiency of the subcutaneous tissue space and promoting the formation of a wider diffusion distribution of extravasated fluid in the subcutaneous area, thereby efficiently removing and draining extravasated fluid. In our case, the hospital did not include hyaluronidase in the procurement catalog or because of its relatively limited clinical application scenarios and limited demand. Additionally, the recommendation of skin testing before use may be another factor that further restricts its clinical application. Wound dressings play a key role in wound care. Besides acting as a physical barrier to prevent injury and infection, alginate dressings also accelerate healing and reduce the risk of infection.^[24]^ Alginate dressings have significant advantages in wound care. They have strong exudative absorbency and can keep the wound moisturized and accelerate wound healing. Calcium ions can be released to stop bleeding and form a physical barrier to prevent infection. It is biocompatible and can relieve pain during dressing changes. It is used mainly for moderate and severe exudative wounds and bleeding wounds.^[25,26]^ Foam dressings usually consist of porous polyurethane foam as the main absorber layer, with a semipermeable membrane backing (such as polyurethane film). This type of dressing exhibits excellent water absorption properties and allows for the permeable exchange of gas and water vapor while forming an effective barrier against bacteria and liquids. In clinical settings, it is used mainly for moderate and severe wounds and is often used as a secondary dressing.^[27]^ Studies have confirmed that it has a significant auxiliary effect on wound repair by promoting the growth of granulation tissue.^[28]^ Hydrocolloid dressings are widely used in clinical practice. Their material is a colloidal gel. Owing to the characteristics of mixing with elasters and adhesives, hydrocolloid dressings have become an important feature that is different from foam dressings and alginate dressings.^[29]^ This feature results in hydrocolloid dressings having significant advantages in terms of fit, comfort, and operation convenience, especially for children with acute and chronic wounds and low-to-moderate exudative wound care.^[30]^ In our case, mechanical debridement combined with high-absorption dressing was used to control exudation in the early stage (first to third debridement), hydrocolloid dressing was used to promote granulation growth in the middle stage (fourth debridement), and growth factors and antibacterial dressings were combined to accelerate epithelialization in the later stage (fifth and sixth debridement), forming a closed-loop management of “debridement-regeneration-repair.” The progressive intervention of wounds from the exudation stage to the repair stage can be achieved by dynamically adjusting the type of dressing. The evaluation at discharge revealed that after 6 rounds of debridement and dressing disposal, the wound exudate volume and necrotic tissue were completely removed, the granulation tissue was properly covered, and the clinical healing criteria were finally met (Table 1).

**Table 1:** Process of wound recovery during debridement after skin extravasation injury.

Time	The area of the trauma	Depth	Color	Exudate volume	Surrounding skin	Smell	N-PASS score
March 29 (the first debridement)	2.5 cm × 1.5 cm	The subcutaneous tissue layer	90% is red granulation tissue and 10% is yellow necrotic tissue	A large amount	Impregnation	There is none	2 points (score)
April 2 (the second debridement)	2.3 cm × 1.5 cm	The subcutaneous tissue layer	95% is red granulation tissue and 5% is yellow necrotic tissue	Medium	Impregnation	There is none	2 points (score)
April 4 (the third debridement)	2.3 cm × 1.5 cm	The subcutaneous tissue layer	100% red granulation tissue layer	Medium	Impregnation	There is none	2 points (score)
April 6 (the fourth debridement)	2.2 cm × 1.3 cm	The subcutaneous tissue layer	95% is red granulation tissue and 5% is yellow necrotic tissue	A small amount	Impregnation	There is none	2 points (score)
April 8 (the fifth debridement)	1.8 cm × 1.0 cm	The subcutaneous tissue layer	95% is red granulation tissue and 5% is yellow necrotic tissue	A small amount	Impregnation	There is none	One point (score)
April 12 (the sixth debridement)	1.2 cm × 0.5 cm	The subcutaneous tissue layer	98% red granulation tissue, with 2% yellow tissue layer remaining	A small amount	Normal	There is none	One point (score)

The N-PASS in the table is the Chinese version of the neonatal pain, agitation and sedation scale (N-PASS).

N-PASS = neonatal pain, agitation and sedation scale.

The guidelines and expert consensus indicate that dynamic assessment for neonatal pain should be conducted and that a stepwise and individualized analgesic regimen should be implemented based on the assessment results.^[31,32]^ The Chinese Expert Consensus on Neonatal Pain Assessment and Analgesia Management (2020 version) recommends that for severe pain,^[17]^ the analgesic management strategy should be based on opioids supplemented with local anesthetics and nondrug interventions. Opioids, such as fentanyl, are mainly used to treat severe pain in neonates.^[33]^ A systematic review revealed that lidocaine gel is effective in reducing perioperative pain scores for neonatal circumcision.^[12]^ Non-nutritive sucking is the most common non-pharmacological intervention for infants in the NICU.^[34]^ The guidelines recommend that the use of glucose water and a curled-up position can effectively relieve procedural pain in neonates.^[14,32,35]^ In our case, the multimodal analgesia regimens of local application (lidocaine gel), opioids (fentanyl), and non-pharmacological interventions (non-nutritional sucking, glucose water, and the curled-up position) for severe neonatal pain were highly consistent with the stepwise and individualized analgesia principles recommended by the guidelines. The decrease in the clinical outcome of the pain score of the patients from N-PASS 2 to 1 and stable vital signs confirmed the effect of fentanyl in the control of severe pain and the synergistic value of lidocaine gel combined with non-pharmacological intervention in improving the safety of analgesia and reducing the risk of operation. These findings suggest that in clinical practice, multimodal analgesia has significant advantages in balancing analgesic efficacy and safety for special groups, such as neonates.

The precise implementation of clinical practice and the effective combination of guidelines and specifications can help ensure that specifications play a crucial role in improving medical quality and ensuring patient safety. Several guidelines have stated that clinical nurses need to learn the content of the guidelines in time,^[20,36]^ pass strict assessments to test the degree of mastery, and accurately implement the standards and specifications in clinical practice. In a study conducted by the department of Neonatology in Australia,^[37]^ the establishment of a vascular expert team and advanced training in the department reduced the Incidence of peripheral intravenous catheter-related complications. However, in this case, the nurse in charge reported that the treatment process following fluid extravasation was not conducted according to standard requirements, and they were unsure of the antidote and alternative therapy to administer. This may be related to the attention given by nursing managers and their handling experience. Jiang et al reported that interdisciplinary teamwork can significantly improve the wound healing rate and reduce the healing time.^[38]^ The interdisciplinary team consisted of plastic surgeons, orthopedists, wound and stoma specialist nurses, and neonatal specialist medical staff. Real-time communication was facilitated by establishing a wound management group chat, and personalized wound treatment plans were subsequently formulated to provide comprehensive and systematic clinical guidance for children.

### 4.1. Enlightenment of nursing

The successful treatment of this case highlighted the important role of interdisciplinary collaboration in dealing with severe neonatal TPN extravasation injury and revealed the key links that need to be strengthened in clinical nursing practice. High-risk early warning and prevention strategies should be strengthened in clinical nursing practice to enhance patient safety. Newborns receiving hypertonic and irritating drugs (especially TPN), especially those who are small for gestational age and have other forms of subcutaneous tissue weakness, should be included in the high-risk group for intravenous therapy. Priority should be given to evaluating and establishing central venous access, choosing a thick, elastic, and easy-to-fix blood vessel puncture site, avoiding the joint area, minimizing the risk of peripheral vascular injury to the greatest extent, and strictly implementing preventive measures. Focus on management is imperative. Second, the ability of emergency treatment for extravasation should be improved. It is required to develop and implement stratified, standard operating procedures for the management of neonatal TPN extravasation and these guidelines must be incorporated into the training and assessment of nurses’ core competence. Thus, it is critical to ensure that nurses possess comprehensive competence in the identification, assessment, and management of neonatal extravasation, and are capable of adapting and implementing context-appropriate intervention protocols according to the specific resources and conditions of their respective healthcare institutions. Finally, the success of this case confirmed that interdisciplinary teams (plastic surgeons, orthopedic surgeons, wound stoma specialist nurses, neonatal specialist medical, and nursing staff) play an indispensable role in complex wound management. During wound treatment, attention should be paid to managing pain, comforting children, alleviating their discomfort, and fostering cooperation with them. Therefore, building and consolidating an interdisciplinary collaboration mechanism, developing a fast response channel, clearly defining roles and responsibilities, sharing information, and continuously improving the treatment process are essential for the efficient healing of children.

**Table:** 

Summary of practice and research recommendations	
What we know thus far:	In small-for-gestational-age infants, venous extravasation may occur when hypertonic total parenteral nutrition is administered. In the clinical classification management of fluid extravasation, patients with grades I and II are mostly treated with observation and conservative treatment, while patients with grades III and IV need active intervention, and it is necessary to control wound infection and promote wound tissue regeneration and repair. Fluid extravasation is mostly treated and evaluated by the department where the adverse event occurs, and interdisciplinary intervention is rarely used. Compound polymyxin ointment has anti-infective ability, and recombinant human epidermal growth factor can promote cell proliferation and differentiation and promote new tissue. Alternating application of the 2 can effectively promote the wound healing of fluid extravasation.
The contents to be studied are:	For severe skin injury caused by infusion extravasation, an interdisciplinary team can integrate the advantages of multidisciplinary treatment to minimize the risk of complications of fluid extravasation. According to the characteristics of the wound, the selection and replacement of the corresponding dressing and the combination of wound healing drugs can accelerate wound healing and achieve good results. In mechanical debridement treatment, it is required to precisely assess the pain level and adopt a multimodal analgesia plan. The pain of the children is relieved and their cooperation is enhanced.
What we can do today:	Newborns (especially premature, small for gestational age infants) who receive hypertonic/irritant medications (such as TPN) should be evaluated and prioritized for establishing central venous access, or choose a thick, elastic peripheral vessel, avoiding the joint site. The treatment process of neonatal TPN extravasation should be formulated and included in the training and assessment of nurses, to strengthen the ability of extravasation identification, assessment and emergency treatment. An interdisciplinary team was established to deal with complex wounds, and the pain management and comfort of children should be paid attention to improve the treatment effect.

## 5. Conclusions

In our patient, severe skin necrosis caused by neonatal TPN extravasation was successfully reversed, and efficient healing was achieved through standardized treatment by an interdisciplinary team. This confirmed the intervention ability of neonatal TPN extravasation-induced skin necrosis but also revealed the core problems of a lack of training systems and insufficient practice standardization in clinical nursing. Further, owing to differences in regional development, there are certain objective limitations in accessing standardized standards in areas with underdeveloped medical resources). The nonstandard treatment administered by primary nurses reflected the weak links of existing training in high-risk drug management, evidence-based practice application, and other dimensions. This case reminds us that it is essential to ensure the safety of clinical infusions by establishing a standardized training system, improving management norms for high-risk drugs, and promoting an interdisciplinary collaboration model.

## Acknowledgments

The authors would like to express their gratitude to all those who participated in this work. Special thanks go to Professor Linqi Zhang, the chairperson of the Pediatric Nursing Committee of the Chinese Nursing Association, for her invaluable guidance and support throughout the project.

## Author contributions

**Data curation:** Danfeng Li, Miaomiao Shi.

**Formal analysis:** Mingli Jiang.

**Investigation:** Guilin Fan.

**Methodology:** Yingyuan Wang.

**Project administration:** Hongyan Wu.

**Resources:** Caixiao Shi, Wenqing Kang.

**Supervision:** Caixiao Shi, Wenqing Kang.

**Validation:** Yingyuan Wang.

**Visualization:** Mingli Jiang, Yanyan Sun.

**Writing – original draft:** Danfeng Li.

**Writing – review & editing:** Hongyan Wu.
